# Mechanical strain regulates the Hippo pathway in *Drosophila*

**DOI:** 10.1242/dev.159467

**Published:** 2018-03-01

**Authors:** Georgina C. Fletcher, Maria-del-Carmen Diaz-de-la-Loza, Nerea Borreguero-Muñoz, Maxine Holder, Mario Aguilar-Aragon, Barry J. Thompson

**Affiliations:** 1 Epithelial Biology Laboratory, The Francis Crick Institute, London NW1 1AT, UK; 2 Apoptosis and Proliferation Control Laboratory, The Francis Crick Institute, London NW1 1AT, UK

**Keywords:** Cell shape, *Drosophila*, Hippo pathway, Mechanosensing, Yorkie

## Abstract

Animal cells are thought to sense mechanical forces via the transcriptional co-activators YAP (or YAP1) and TAZ (or WWTR1), the sole *Drosophila* homolog of which is named Yorkie (Yki). In mammalian cells in culture, artificial mechanical forces induce nuclear translocation of YAP and TAZ. Here, we show that physiological mechanical strain can also drive nuclear localisation of Yki and activation of Yki target genes in the *Drosophila* follicular epithelium. Mechanical strain activates Yki by stretching the apical domain, reducing the concentration of apical Crumbs, Expanded, Kibra and Merlin, and reducing apical Hippo kinase dimerisation. Overexpressing Hippo kinase to induce ectopic activation in the cytoplasm is sufficient to prevent Yki nuclear localisation even in flattened follicle cells. Conversely, blocking Hippo signalling in *warts* clones causes Yki nuclear localisation even in columnar follicle cells. We find no evidence for involvement of other pathways, such as Src42A kinase, in regulation of Yki. Finally, our results in follicle cells appear generally applicable to other tissues, as nuclear translocation of Yki is also readily detectable in other flattened epithelial cells such as the peripodial epithelium of the wing imaginal disc, where it promotes cell flattening.

## Abstract (precis)

**Highlighted Article:** *Drosophila* Yorkie can sense physiological mechanical strain forces via the canonical Hippo pathway in different epithelial tissues.

## INTRODUCTION

The Hippo signalling pathway was discovered in *Drosophila* as being essential to restrict cell proliferation in growing tissues (reviewed by Badouel et al., 2009b; Halder and Johnson, 2011; Harvey and Hariharan, 2012; Pan, 2010). The core Hippo (Hpo/MST)-Warts (Wts/LATS) kinase cassette is activated by the Crumbs-Expanded (Crb-Ex) and Merlin-Kibra (Mer-Kib) protein complexes at apical cell-cell junctions (Badouel et al., 2009a; Baumgartner et al., 2010; Chen et al., 2010; Genevet et al., 2010; Hamaratoglu et al., 2006; Ling et al., 2010; Robinson et al., 2010; Su et al., 2017; Yu et al., 2010). Another apical cell-cell junction protein, Echinoid, may also contribute to activating Hpo-Wts signalling in *Drosophila* (Yue et al., 2012). Furthermore, Wts activity is inhibited by E-cadherin-associated proteins such as Ajuba (Jub) and Zyxin (Das Thakur et al., 2010; Gaspar et al., 2015; Jagannathan et al., 2016; Rauskolb et al., 2011; Rauskolb et al., 2014), and by Dachsous-cadherin-associated proteins, such as Dachs, Mib or Riq (Degoutin et al., 2013; Mao et al., 2006; Vrabioiu and Struhl, 2015). Once activated, Wts directly phosphorylates the key nuclear effector Yki (called YAP and TAZ in mammals) on conserved serine residues to induce binding to 14-3-3 proteins and retention in the cytoplasm (Dong et al., 2007; Huang et al., 2005; Oh and Irvine, 2008, 2009). Mutation of Wts, or mutation of multiple target serine residues in Yki (3SA) or YAP (5SA) is sufficient to induce nuclear translocation of Yki or YAP, which co-activates the DNA-binding transcription factor Scalloped/TEAD to drive target gene expression (Dong et al., 2007; Huang et al., 2005; Oh and Irvine, 2008, 2009).

How Yki, YAP and TAZ are physiologically regulated is still poorly understood. In mammalian cell culture, YAP and TAZ act as mechanotransducers, being cytoplasmic in densely packed cells and becoming strongly nuclear when cultured cells are stretched flat (Benham-Pyle et al., 2015; Dupont et al., 2011; Zhao et al., 2007). Interestingly, strong nuclear localisation of YAP depends on formation of basal F-actin stress fibres and basal Integrin-Src signalling in cultured cells (Elbediwy et al., 2016; Elosegui-Artola et al., 2016; Kaneko et al., 2014; Kim and Gumbiner, 2015; Tang et al., 2013; Wada et al., 2011). Src can directly phosphorylate YAP on three tyrosines in its transcriptional activation domain to promote YAP activity (Li et al., 2016). However, it remains unclear whether Integrin-Src signalling acts directly on YAP or via the canonical Hippo signalling pathway (Si et al., 2017).

In *Drosophila*, Yki transcriptional activity appears to be elevated in mildly circumferentially stretched cells of the developing larval wing imaginal disc, as indicated by upregulation of the *expanded.lacZ* (*ex.lacZ*) reporter gene in stretched cells (Fletcher et al., 2015; Mao et al., 2013). However, the degree of cellular stretching in the wing disc is not strong enough to induce an obvious nuclear localisation of Yki, which instead remains mostly cytoplasmic in most wing cells (Oh and Irvine, 2008). Thus, there is still no convincing evidence that physiological stretch forces can regulate canonical Hippo signalling to drive Yki to the nucleus *in vivo.* Despite the lack of a compelling *in vivo* system to study mechanical regulation of Yki subcellular localisation, different models have been proposed for how Yki might respond to force. We previously proposed that the canonical upstream components of the Hippo pathway, such as apical Crb-Ex and Mer-Kib, would become diluted upon stretching of the apical domain, reducing their ability to cluster and induce transactivation of Hippo kinase (Fletcher et al., 2015). An alternative model proposed that cytoskeletal tension acts through a Rho-Rok-Myo-II pathway to promote localisation of Ajuba to adherens junctions, where it directly recruits and inhibits Wts kinase (Pan et al., 2016; Rauskolb et al., 2014). Finally, there is not yet good evidence for a physiological role for Integrin-Src signalling in activating Yki, although overexpression of Src can induce Yki target gene expression via an indirect mechanism involving cytoskeletal changes and JNK activation (Fernandez et al., 2014). Proof of any of these models requires a demonstration that physiological cellular stretching is sufficient to affect the proposed mechanism and that the effects of stretching on Yki localisation can be reversed by manipulating the proposed pathway.

Here, we show that *Drosophila* Yki can sense physiological mechanical strain forces via the canonical Hippo pathway. We propose that stretching of the apical domain dilutes the concentration of apical Hippo pathway components, reducing the dimerisation of the Hippo kinase to downregulate Hippo signalling and thereby activate Yki.

## RESULTS

**Fig. 1.**
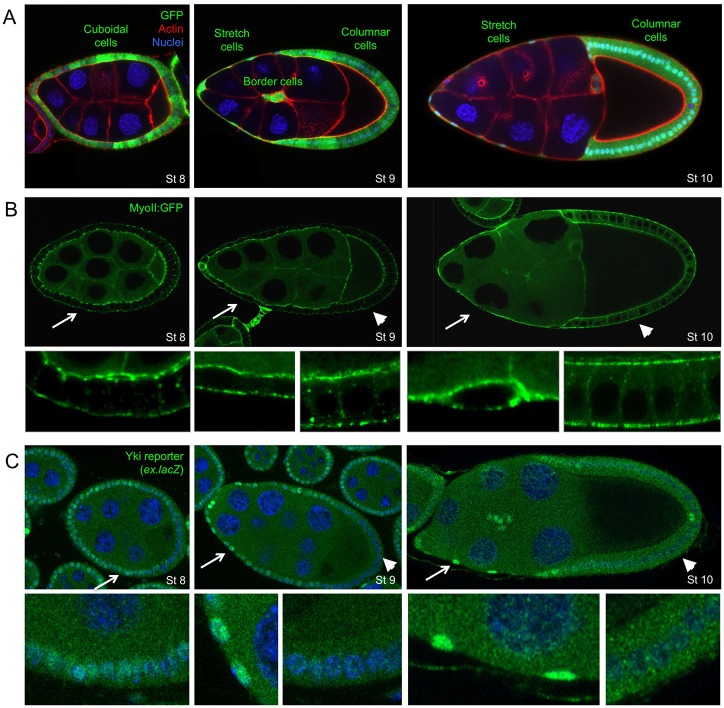
**Yki target gene expression is induced in stretch cells and repressed in columnar cells.** (A) *Drosophila* egg chambers at stages 8, 9 and 10 of oogenesis. The largest cell is the oocyte, which is fed by the polyploid nurse cells. Surrounding the germline cells are the somatic follicular epithelial cells (green, GFP^+^). At stage 8, all follicle cells are cuboidal. At stage 9, anterior follicle cells become stretched while posterior follicle cells remain cuboidal or columnar. By stage 10, anterior follicle cells are completely stretched flat, whereas posterior columnar cells surround the oocyte. Note also migration of border cells during stage 9. (B) The level of endogenously tagged Myosin-II:GFP does not change during formation of stretch cells or columnar cells. The actomyosin cortex labelling reveals the degree of flattening of stretch cells versus columnar cells. (C) A Yki reporter gene (*expanded.lacZ)* is upregulated in stretch cells and downregulated in columnar cells. The two polar cells also show increased *ex.lacZ* expression. DAPI is shown in blue and phalloidin is shown in red. Arrows and arrowheads indicate the regions enlarged in the panels below.

We first sought to investigate the regulation of Yki in the ovarian follicular epithelium of *Drosophila*, where growth of the egg chamber is suggested to cause a dramatic flattening of a group of previously cuboidal anterior follicle cells called the ‘stretch cells’ (Horne-Badovinac and Bilder, 2005; Kolahi et al., 2009). An attraction of this model system is that the posterior follicle cells that contact the oocyte become columnar, rather than flattening, and therefore act as an internal control (Fig. 1A). Cortical Myosin II staining reveals the dramatic change in cell shape, but does not increase in intensity in stretch cells relative to columnar cells, suggesting that flattening involves a large increase in mechanical strain (cellular shape change) without a correspondingly large increase in mechanical stress (external tension forces, which normally induce myosin II contractility) (Fig. 1B). We find that the activity of Yki, as measured by the reporter gene *ex.lacZ*, is initially moderately active in all follicle cells at early stages (Fig. 1C). Upon flattening of the stretch cells at stage 9 of development, *ex.lacZ* expression becomes elevated in the stretch cells while becoming repressed in the posterior follicle cells that contact the oocyte and pack densely to acquire a columnar cell shape (Fig. 1C). An interesting exception is the two polar cells, which also upregulate *ex.lacZ* (Fig. 1C). These findings demonstrate physiological induction of Yki activity as mechanical flattening of epithelial cells takes place during follicular development.

**Fig. 2.**
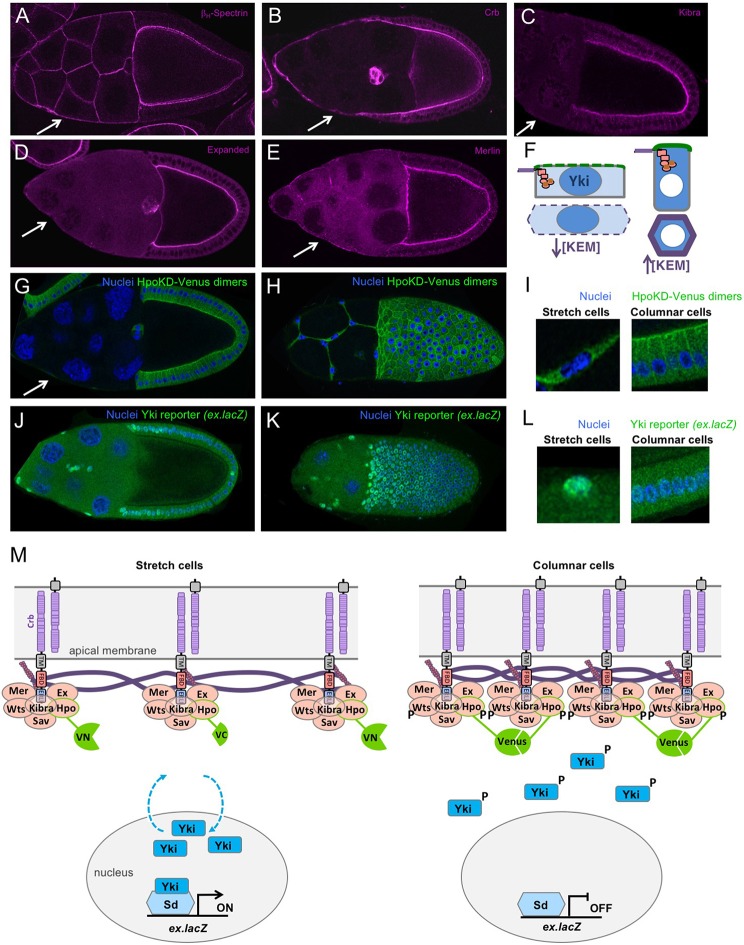
**Canonical upstream Hippo pathway components concentrate apically in columnar cells and are diluted in stretch cells to control Hippo kinase dimerisation and signalling.** (A-E) The upstream Hippo pathway components, β-Heavy Spec (A), Crb (B), Kib (C), Ex (D) and Mer (E) strongly label the apical membrane domain of columnar follicle cells but become less concentrated in stretch cells. (F) Schematic diagram showing dilution of Kib, Ex and Mer proteins upon stretching of the apical domain. Green represents the apical domain. Purple/orange represents the apical-junctional ring of Crb-Ex and Mer-Kib complexes. Blue represents Yki localisation. (G-I) Measurement of Hippo kinase dimerisation with a split-Venus reporter reveals strong apical membrane signal in columnar cells and weak membrane signal in stretch cells. Both columnar and stretch cells have some cytoplasmic background Venus signal. G is a cross-section. H is a surface view. I contains magnified views of G and H. (J-L) Yki reporter gene expression is inversely correlated with Hippo kinase activation at the plasma membrane (compare with G-I). J is a cross-section. K is a surface view. L contains magnified views of G and H. (M) Schematic diagram showing dilution of upstream Hippo pathway complexes, reduced Hpo-Wts phosphorylation, reduced Yki phosphorylation, and increased Yki nuclear localisation and transcriptional activation upon stretching of the apical membrane domain. Measurement of Hpo dimerisation via the split-Venus BiFC reporter is diagrammed. Arrows indicate stretch cells.

To investigate the mechanism of Yki regulation in stretch cells, we analysed the localisation of the apical β-HeavySpectrin (Karst; Kst) as well as several key upstream regulators of the Hippo pathway: Crb, Mer, Ex and Kib. Each of these proteins remains apically localised in the columnar follicle cells but becomes strongly diluted across the apical surface of stretch cells (Fig. 2A-F). Accordingly, a Hippo kinase bimolecular fluorescence dimerisation sensor [HpoKD-Venus BiFC (Deng et al., 2013)] is active at the apical domain of columnar follicle cells contacting the oocyte but not active at the plasma membrane of stretch cells (Fig. 2G-I). This same sensor fails to homodimerise in S2 cells in culture, which lack an apical domain (Deng et al., 2013). Dilution of apical proteins in stretch cells is likely to account for the reduced Hippo kinase activation, because overexpression of Crb or Ex is sufficient to activate the Hippo dimerisation reporter in all follicle cells (Fig. S1). There is also a mild contribution from lateral Tao-1 kinase (Fig. S2). Importantly, the dilution of apical proteins and Hippo kinase dimerisation in stretch cells correlates with increased Yki activity, as measured with *ex.lacZ* expression (Fig. 2J-L). However, we note that some residual *ex.lacZ* expression perdures in a gradient of columnar follicle cells located adjacent to the stretch cells, consistent with the fact that these cells arrive in temporal sequence to change from a cuboidal to a columnar shape (Fig. 2J,K). These findings suggest a simple model of stretch-induced Yki activation via reduced apical Hippo dimerisation (Fig. 2M), which can be elaborated to incorporate spatially distinct Crb-Ex and Kib-Mer complexes (Fig. S3) (Su et al., 2017).

**Fig. 3.**
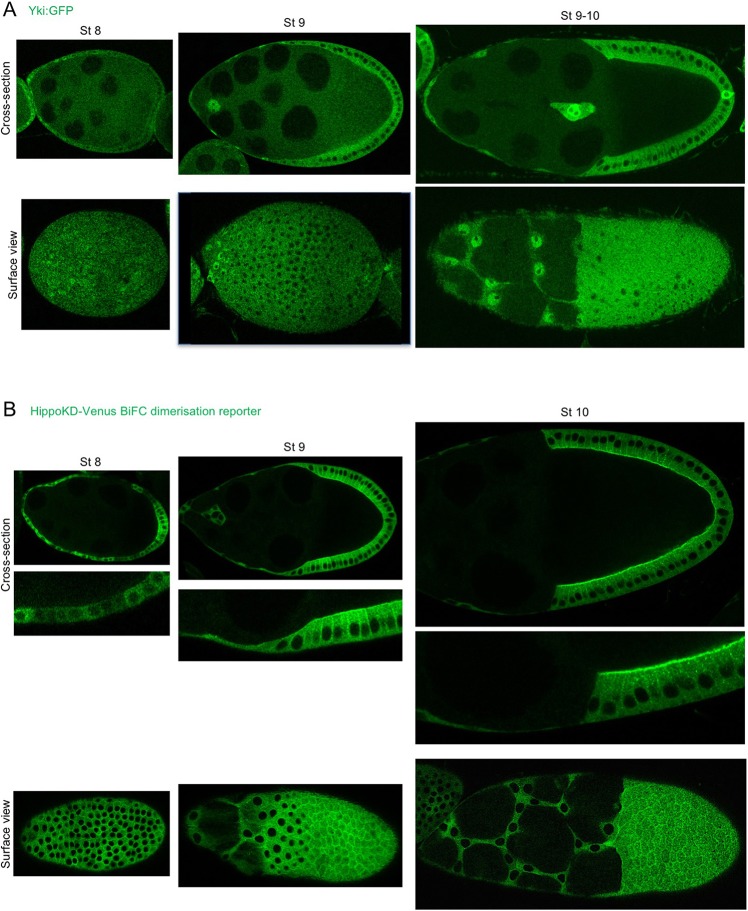
**Yki translocates to the nucleus in stretch cells and to the cytoplasm of columnar cells, inversely correlating with Hippo dimerisation at the apical plasma membrane.** (A) Endogenous Yki with a GFP tag was visualised in the views shown at different stages of oogenesis. At stage 8, Yki:GFP is predominantly cytoplasmic in the cuboidal epithelium, but at the anterior where the cells start to flatten Yki:GFP is found in the nucleus. During stage 9, Yki:GFP can clearly be found in the nucleus of stretch cells and in the cytoplasm of columnar cells. This pattern is even more pronounced at stage 10. (B) The HippoKD-Venus dimerisation reporter was visualised in the views shown over different stages of oogenesis. At stage 8, a weak Hippo dimerisation signal is observed with an apical signal apparent in the posterior epithelium. At stages 9 and 10, a clear apical and lateral signal can be seen in columnar cells, whereas in stretch cells there is a faint cytoplasmic signal.

**Fig. 4.**
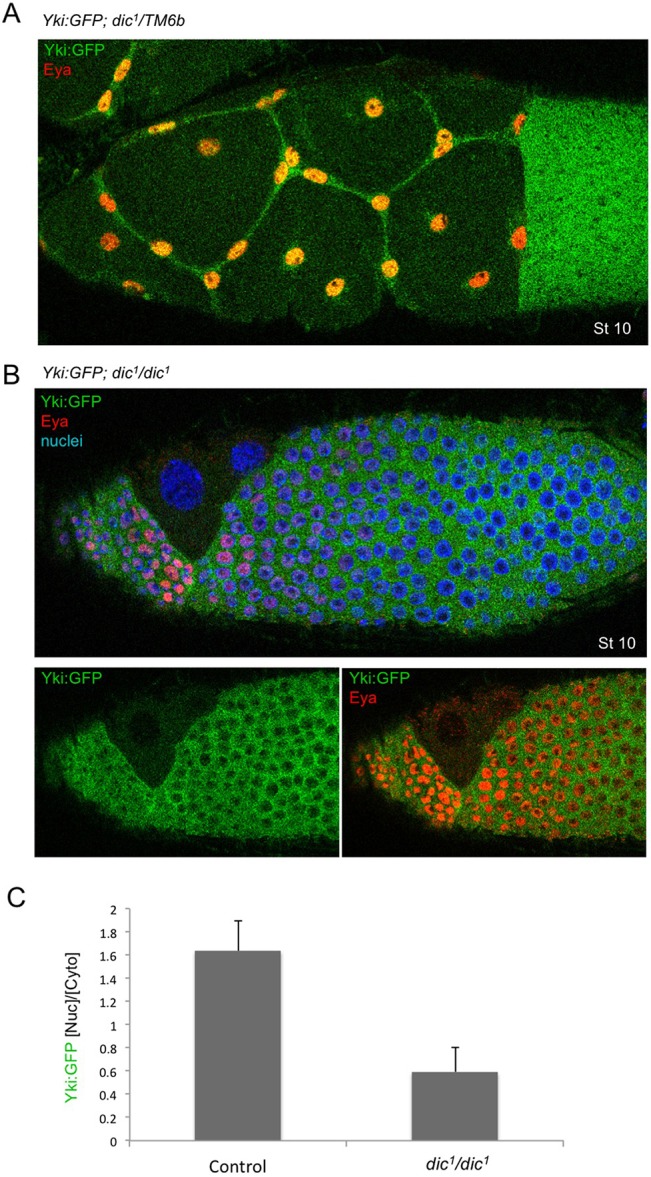
***Dicephalic* mutants disrupt stretch cell flattening, but not cell fate, and reduce nuclear Yki:GFP*.*** (A) Control and (B) *Dicephalic (dic^1^/dic^1^)* mutant stage 10 egg chambers (Eya staining is in red, DAPI staining is in blue) show that the less stretched cells in the *dic* mutant have less nuclear Yki:GFP than the control. (C) Quantification of the ratio of nuclear to cytoplasmic Yki:GFP (*n*=12). Data are mean±s.d. The difference is statistically significant (*P*<0.001).

To analyse the subcellular localisation of Yki in real time, we generated a *Yki:GFP* knock-in line that is homozygous viable and fertile (Fig. 3A). We find that the Yki:GFP protein localises to the nucleus upon flattening of stretch cells, while remaining cytoplasmic in columnar follicle cells, inversely correlating with apical Hippo dimerisation (Fig. 3A,B; Fig. S4). In order to test whether nuclear Yki:GFP localisation depends on mechanical flattening of stretch cells, rather than being a consequence of stretch cell fate determination, we examined Yki:GFP in *Dicephalic* (*dic^1^*) mutants, in which adhesion between the germ cells and somatic follicle cells is disrupted and prevents stretch cell flattening (McCaffrey et al., 2006). We found that Yki:GFP fails to localise to the nucleus in *dic^1^* mutants, despite the fact that stretch cell fate is properly specified (as revealed by expression of the transcription factor eyes-absent or Eya) (Fig. 4). These findings support the notion that Yki:GFP responds to the morphology of stretch cells versus columnar cells, rather than any other signalling mechanism that controls stretch cell fate.

**Fig. 5.**
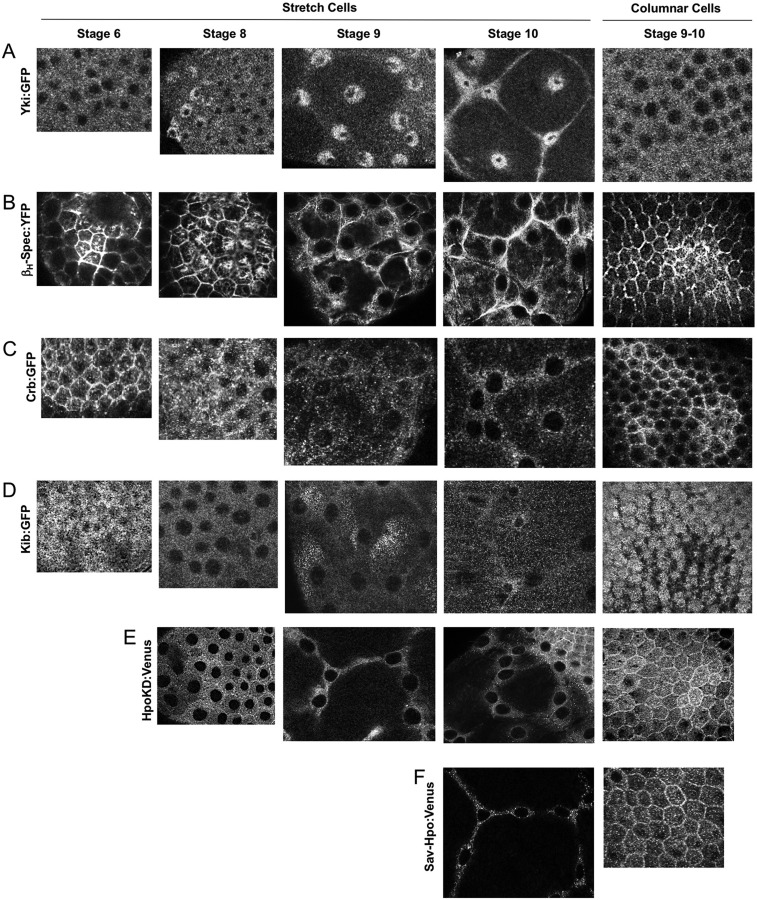
**High-resolution imaging of Yki translocation and Hippo pathway component dilution as the apical domain becomes diluted in stretch cells compared with columnar cells.** Top-down views of stretch cells and columnar cells at progressive stages of oogenesis: (A) Yki:GFP, (B) β_H_-Spectrin:YFP, (C) Crb:GFP, (D) Kib:GFP, (E) HpoKD-Venus dimers and (F) Sav-HpoKD-Venus dimers.

We next sought to examine the spatial-temporal relationship between nuclear localisation of Yki:GFP and dilution of apical Hippo pathway components at high resolution. We examined Yki:GFP, β-HeavySpectrin-YFP, Crb:GFP and Kib:GFP in stretch cells as they undergo mechanical strain that converts them from cuboidal shape (at stage 6) through to a highly flattened shape (at stage 10). We find that increasing nuclear Yki:GFP correlates precisely with dilution of all three apical proteins (Fig. 5A-D, Fig. S4). In contrast, Yki:GFP remains cytoplasmic in columnar cells, which have highly concentrated apical complexes. We note that Crb:GFP is mostly apical-junctional, whereas Kib:GFP is localised to the apical-medial region as previously reported (Su et al., 2017). Importantly, the Hpo dimerisation sensor (as well as an alternative Hpo-Sav dimerisation sensor) is active at both the apical-junctional and apical-medial zone of columnar epithelial cells (Fig. 5E,F). These findings suggest that Crb-Ex and Kib-Mer complexes both contribute to Hippo kinase activation in columnar cells to drive Yki to the cytoplasm, whereas dilution of the apical domain reduces Hippo dimerisation to drive nuclear Yki localisation in stretch cells.

**Fig. 6.**
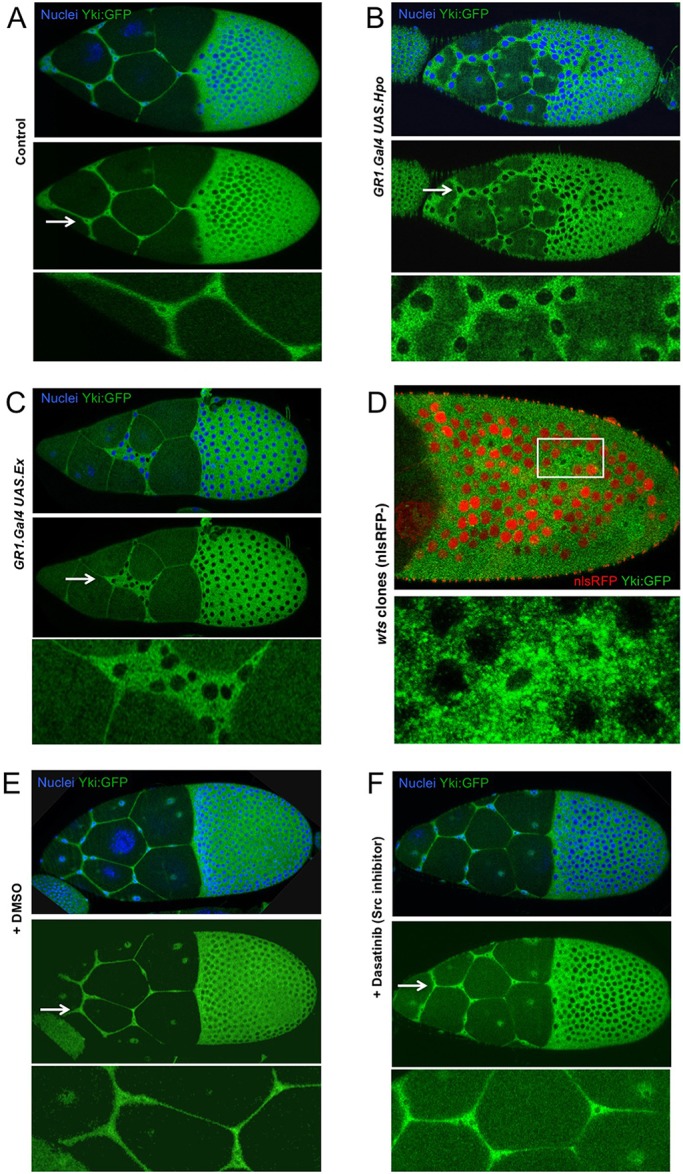
**Canonical Hippo signalling is necessary and sufficient to control the nuclear localisation of Yki:GFP in follicle cells, whereas a Src family kinase inhibitor has no effect.** (A) Endogenously tagged Yki:GFP localises to the nucleus in stretch cells and to the cytoplasm in columnar cells. (B) Overexpression of Hippo kinase (Hpo) with GR1.Gal4 in follicle cells causes Yki:GFP to relocalise to the cytoplasm in all cells. (C) Overexpression of Ex with GR1.Gal4 in follicle cells causes Yki:GFP to relocalise to the cytoplasm in all cells. (D) Mutation of Wts in clones marked by the absence of nuclear RFP (red) causes nuclear localisation of Yki:GFP in columnar cells. Boxed area is magnified in the panel below. (E) DMSO-treated control shows normal localisation of Yki:GFP in stretch cells and columnar cells. (F) Inhibition of Src family kinases with dasatinib has no effect on the localisation of Yki:GFP in either stretch cells or columnar cells. Arrows indicate the regions enlarged in the panels below.

To test this model, we examined whether the canonical Hippo pathway is the sole regulator of Yki:GFP localisation in follicle cells. We find that activation of canonical Hippo signalling by overexpression of either Ex or Hpo is sufficient to relocalise Yki:GFP to the cytoplasm in stretch cells (Fig. 6A-C) without affecting stretch cell fate (Fig. S5A). Clonal analysis of Hpo overexpression indicates that this is a cell-autonomous effect (Fig. S5B). Opposingly, inactivation of Hippo signalling by mutation of the *wts* gene is sufficient to induce nuclear localisation in columnar follicle cells (Fig. 6D; Fig. S6). In contrast, inactivation of the possible alternative mechanosensor Src by treatment with the Src inhibitor dasatinib or by expression of RNAi in all follicle cells has no effect on Yki:GFP localisation or Yki target gene expression (Fig. 6E,F and Fig. S7). During wing growth, Src depletion does not affect cell proliferation at larval stages or wing expansion during early metamorphosis (Fig. S8). In both tissues, the wing and the ovary, overexpression of Src also appears to affect Yki indirectly via disruption of epithelial polarity (Fig. S9). These findings show that Src activity is dispensable for regulation of Yki, whereas mechanical regulation of canonical Hippo signalling is necessary and sufficient to control nuclear localisation of Yki in *Drosophila* follicle cells.

**Fig. 7.**
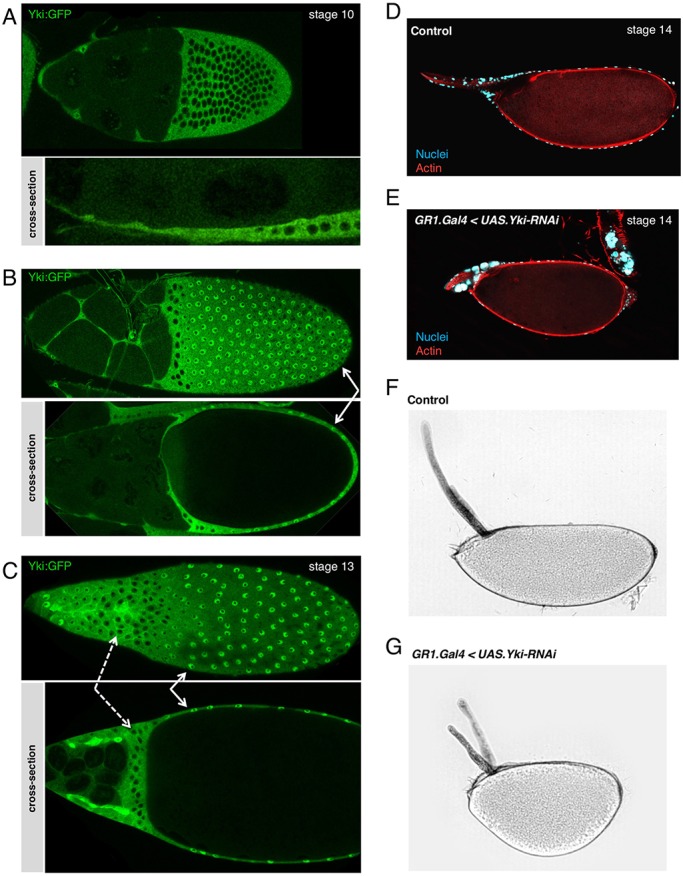
**Nuclear localisation of Yki:GFP occurs in mechanically stretched cells throughout *Drosophila* development.** (A) In stage 10 egg chambers, Yki:GFP accumulates in the nucleus in extremely flat follicle cells in the anterior half of the egg chamber (stretch cells), and is exclusively cytoplasmic in columnar posterior follicle cells (main body follicle cells). (B) In stage 11 egg chambers, Yki:GFP accumulates in the nuclei of stretch cells and in the progressively flatter posterior main body follicle cells (white arrows). However, it remains cytoplasmic in the non-stretched follicle cells in the middle. (C) In stage 13 egg chambers, Yki:GFP localises in the nuclei of main body follicle cells at the posterior (white arrows), and in the precursors of the dorsal appendages at the anterior tip of the egg chamber, which are also flattening. Non-stretched centripetal cells accumulate Yki:GFP in the cytoplasm (dashed arrows). (D) Control stage 14 egg chamber. Nurse cell nuclei have disappeared, only the nuclei belonging to the muscle sheath that cover the egg are present around the dorsal appendages. (E) Stage 14 *TJ.G4, UAS.Yki-RNAi* egg chamber in which the nurse cell nuclei have failed to be degraded. (F) Bright-field image of a control egg mature egg. (G) Bright-field image of a *TJ.G4 UAS.Yki-RNAi* mature egg showing reduced extension in the anterior-posterior axis and shorter dorsal appendages.

To extend our findings in stretch cells, we examined later stages of oogenesis, when enlargement of the oocyte causes the entire follicle cell epithelium to become stretched flat around the oocyte. We find that Yki:GFP translocates from the cytoplasm of initially columnar follicle cells at stage 10 to the nucleus of flattened cells by stages 11-13 (Fig. 7A-C). These results confirm that Yki:GFP is responding to mechanical strain (a change in cell shape) rather than any other differences between follicle cells.

We next sought to consider what the function of Yki might be in *Drosophila* follicle cells. One possible role for a sensor of mechanical strain would be to drive a cellular response that enables cells to accommodate the morphogenetic change as they flatten. To test this notion, we examined the consequence of removing Yki from the follicle cell epithelium during the stages of morphogenetic flattening. We find that silencing of *Yki* expression in follicle cells by RNAi prevents the full dumping of the nurse cells into the oocyte from stages 11-14, such that the oocyte fails to properly expand and elongate, resulting in rounded eggs (Fig. 7D-G). These findings suggest that Yki activity may be required in follicle cells as they flatten to accommodate the full expansion of the oocyte.

**Fig. 8.**
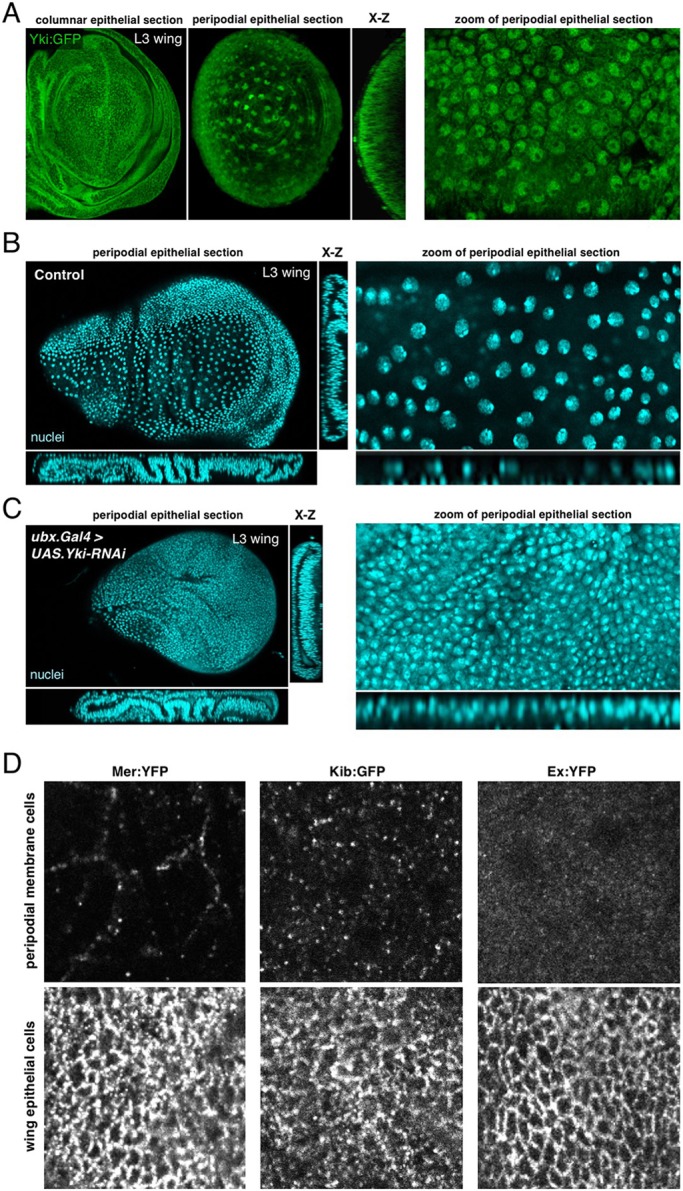
**Nuclear localisation of Yki occurs in the peripodial epithelium of the developing wing and is required for cell flattening and tissue expansion.** (A) In third instar (L3) wing discs, Yki:GFP accumulates in the nuclei of the extremely stretched cells that are the peripodial membrane, but is mostly cytoplasmic in the highly columnar wing epithelial cells. (B) Control L3 wing discs stained with DAPI (blue). Wing cross-sections (left) and high magnifications of peripodial membrane cells (right). (C) *Ubx.Gal4*, *UAS.Yki-RNAi* L3 wing discs stained with DAPI (blue) showing reduced flattening of the peripodial membrane and a smaller wing disc area, as wing epithelial cells have been displaced to the peripodial membrane region to compensate for the reduction of tissue area. Wing cross-sections (left), and high magnifications of peripodial membrane cells (right). (D) Distribution of Mer, Kib and Ex in peripodial epithelial cells versus the underlying columnar epithelia of the third instar wing imaginal disc. Note the strong dilution of these complexes in the peripodial epithelial cells.

To extend our findings beyond oogenesis to other tissues, we examined Yki:GFP in flattening peripodial epithelial cells during wing development. We find that Yki:GFP once again becomes nuclear in the flattened peripodial epithelium, but not the underlying columnar epithelial cells (Fig. 8A). As in the follicular epithelium, expression of *Yki-RNAi* in the peripodial cells causes a failure of these cells to stretch flat over the growing columnar epithelium (Fig. 8B,C). Finally, as in the follicular epithelium, dilution of apical Hippo pathway components coincides with apical domain stretching and Yki translocation to the nucleus (Fig. 8D). These results further confirm that nuclear localisation of Yki is a general response to mechanical strain acting upon the apical domain of epithelial cells rather than one specific to a particular cell type. They also indicate that one overlooked function of Yki in mitotically inactive cells is to facilitate the elastic stretching response to mechanical strain.

## DISCUSSION

Our results demonstrate that physiological mechanical strain forces are sensed via the canonical Hippo pathway in *Drosophila* epithelia*.* Flattening of the so-called ‘stretch cells’ of the follicle cell epithelium is associated with Yki nuclear localisation and Yki-target gene expression (Figs 1–3). *Drosophila* Yki is controlled exclusively by the canonical Hpo-Wts signalling pathway, which is activated primarily at the apical domain of columnar epithelial cells by the Crb-Ex and Kib-Mer complexes (Baumgartner et al., 2010; Genevet et al., 2010; Hamaratoglu et al., 2006), which associate with the apical Spectrin cytoskeleton to form apical-lateral cell-cell junction complexes (Chen et al., 2010; Deng et al., 2015; Fletcher et al., 2015; Ling et al., 2010; Robinson et al., 2010; Yue et al., 2012). Our results show that the concentration of the Crb-Ex and Kib-Mer complexes and activation of Hippo dimerisation at the apical domain of follicle cells is reduced in stretch cells, leading to nuclear localisation of Yki (Figs 2-6 and Figs S1-S6). In addition, stretch cells also lose their lateral domains, where some Hippo dimerisation can also be activated by lateral spectrins and the Tao-1 kinase (Fig. S2). Notably, Src signalling does not appear to contribute to activation of Yki in stretch cells (Figs S7 and S8). Accordingly, overexpression of Src42 appears to have an indirect effect on Yki target genes via disruption of epithelial polarity (Fig. S9). Thus, mechanical control of the canonical Hippo pathway is a primary mechanism underpinning the physiological regulation of Yki during development.

One important function for stretch-induced activation of Yki in follicle cells is to promote cell flattening, such that inhibiting Yki activity causes a mild defect in the spreading of stretch cells at stage 10, and at later stages interferes with final size and shape of the egg (Fig. S1G-I and Fig. 7). A similar function for Yki is evident in the developing wing, where flattening of the peripodial cells involves nuclear localisation of Yki, which is then required to facilitate this morphogenetic change (Fig. 8). Further work will be necessary to characterise the transcriptional target genes that mediate the cellular response to mechanical strain. These findings indicate the generality of stretch-induced Yki activation in different epithelia. However, we note that compression-induced apoptosis in other tissues does not appear to involve Yki inhibition, suggesting that other signalling pathways are more sensitive to the mechanical stress forces involved in cellular compression (Levayer et al., 2016).

Notably, the peripodial epithelium is not a proliferating tissue, similar to the follicle cell epithelia at the late stages we have examined, which enables these cells to undergo dramatic flattening in response to mechanical strain. In proliferating epithelial tissues, such as human epithelia in culture, the response to increased mechanical strain forces is to increase YAP activity and cell proliferation (Benham-Pyle et al., 2015). It will therefore be interesting to determine whether physiological stretch-induced proliferation also occurs via the Hippo pathway in *Drosophila* as it appears to in human epithelial cells in culture.

Interestingly, the observed inhibition of YAP in tightly packed human epithelial cells leads to a well-known outcome: inhibition of cell proliferation (Benham-Pyle et al., 2015; Zhao et al., 2007). In non-proliferating follicle cells, we find that physiological inhibition of Yki in packed columnar follicle cells at stage 10 of oogenesis also appears to have a detectable functional consequence. Translocation of Yki:GFP to the cytoplasm (Fig. 3A) correlates with a sudden downregulation of Crb at this moment in development, suggestive of a negative-feedback loop (Sherrard and Fehon, 2015b). Our data indicate that the physiological activation of Hippo signalling and reduced Yki activity in columnar cells at this stage is causative for the downregulation of Crbs (Fig. S10), consistent with previous evidence that Yki promotes expression of Crb, Ex and other apical proteins (Genevet et al., 2009; Hamaratoglu et al., 2009), as well as with a possible role of upstream Hippo pathway components in promoting Crb endocytosis (Maitra et al., 2006; Sherrard and Fehon, 2015a).

Thus, mechanical strain forces that enlarge the apical domain may be sensed via apical dilution, reduced Hippo signalling, and active Yki to promote expression of apical proteins and thus help sustain the apical domain in response to stretch. Conversely, concentration of the apical domain in densely packed columnar epithelial cells induces Hippo signalling to downregulate apical proteins such as Crb. Thus, in different contexts, mechano-sensing via the Hippo pathway can be employed to regulate either cell proliferation or cellular morphogenesis, or simply to help promote apical domain homeostasis.

## MATERIALS AND METHODS

### *Drosophila* genetics

Mitotic clones in follicle cells were generated using the FLP/FRT system and were marked by the absence of GFP or RFP. Third instar larvae (L3) or newly eclosed adults were heat-shocked once at 37°C for 1 h and dissected either 4 days later or 4 days after eclosion, respectively. Expression of UAS- transgenes in follicle cells was achieved with either the actin ‘flip-out’ Gal4 system, *Traffic Jam-Gal4* (*TJ.Gal4*) or *GR1-Gal4* drivers, and in wing imaginal disc cells with the *nubbin.Gal4* (*nub.Gal4*) driver or *Hedgehog-Gal4* (*hh.Gal4*) driver in the posterior compartment*.* Depletion of Yki in the wing peripodial cells was driven by *Yki-RNAi* using the *Ubx-Gal4* driver; crosses were maintained at 18°C to inhibit *Gal4* expression using the *tubgal80* system, and shifted to 29°C from late first instar to third instar larvae. Fly crosses were kept at a temperature of 25°C or 18°C when necessary. For the experiments on adult flies, wings were collected from adult females 11-12 days post-egg laying and were mounted in Hoyer's mounting medium; ovaries were dissected and immunostained from adult females 4 days after eclosion. Larvae were dissected at stage L3 of development, and pupal wings were dissected at 4 and 7 h after puparium formation. Dasatanib treatment of ovaries expressing *Yki:GFP* was performed by isolating egg chambers and culturing them as described (Khanal et al., 2016) with 20 μm dasatanib (Stratech) or DMSO control for 2 h. After treatment, samples were fixed and processed normally for imaging.

CRISPR/Cas9 genome editing was used to tag the C-terminus of Yki with eGFP. 100 ng/µl of guide RNA plasmid (pCFD3, Addgene 49410, encoding the gRNA 5′-TCAGGTTTGTGGGAAGACGG-3′), plus 500 ng/µl of a homologous recombination repair template plasmid, were co-injected into nos-Cas9 embryos. The repair template contained 4.5 kb genomic DNA from the Yki locus (centred around the stop codon), with eGFP inserted in-frame after the final amino acid of Yki and before its stop codon. The gRNA target sequence in the repair template was mutagenised to prevent re-cutting of correctly targeted alleles. The resulting knock-in allele is homozygous viable.

### *Drosophila* genotypes

The *Drosophila* genotypes are as follows: Fig. 1A, *yw hs.flp; actin.FRT.STOP.FRT.Gal4 UAS.GFP*; Fig. 1B, *yw cv sqh^Ax3^; P{sqh-GFP}*; Fig. 1C: *ex^lacZ^/Cyo*; Fig. 2A-E, *w^iso^*; Fig. 2G-I, *TJ.Gal4, UAS.Hippo^Kinase-dead^VenusC; UAS.Hippo^Kinase-dead^VenusN/Sm6-Tm6b* (Deng et al., 2013); Fig. 2J-L, *ex^lacZ^/Cyo*; Fig. 3A, *Yki:GFP*; Fig. 3B: *Traffic Jam.Gal4, UAS.Hippo^Kinase-dead^VenusC; UAS.Hippo^Kinase-dead^ VenusN/Sm6-Tm6b*; Fig. 4A, *Yki:GFP, dic^1^/Tm6b* (Bloomington 4223); Fig. 4B, *Yki:GFP, dic^1^/dic^1I^*; Fig. 5A, *Yki:GFP*; Fig. 5B, *Karst:YFP*; Fig. 5C, *Crb:GFP*; Fig. 5D, *Kib:GFP* (Su et al., 2017); Fig. 5E, *TJ.Gal4, UAS.Hippo^Kinase-dead^VenusC; UAS.Hippo^Kinase-dead^VenusN/Sm6-Tm6b*; Fig. 5F, *TJ.Gal4, UAS.Hippo^Kinase-dead^VenusC; UAS.Sav VenusN/Sm6-Tm6b* (Deng et al., 2013); Fig. 6A, *Yki:GFP*; Fig. 6B, *UAS.Hpo; +/Yki:GFP ; GR1.G4/+*; Fig. 6C, *UAS.Ex; +/Yki:GFP ; Gr1.G4/+*; Fig. 6D, *Yki:GFP /+; FRT82 nlsRFP/FRT82 wtsX1*; Fig. 6E, *Yki:GFP*; Fig. 6F, *Yki:GFP*; Fig. 7A-C, *Yki:GFP*; Fig. 7D, *Yki:GFP; UAS.Yki-RNAi/TM6B*; Fig. 7E, *Yki:GFP; GR1.Gal4/UAS.Yki-RNAi* (Bloomington 34067); Fig. 7F, *Yki:GFP; UAS.Yki-RNAi/TM6B*; Fig. 7G, *Yki:GFP; GR1.Gal4/UAS.Yki-RNAi* (Bloomington 34067); Fig. 8A, *Yki:GFP*; Fig. 8B, *+,UAS.Yki-RNAi/ Sm6-Tm6b*; Fig. 8C, *tubgal80/+; Ubx.Gal4/UAS.Yki-RNAi* (Bloomington 34067); and Fig. 8D, *Mer:YFP, Kib:GFP, Ex:YFP* (Su et al., 2017).

The genotypes of the flies used in the supplementary figures can be found in Table S1.

### *Drosophila* immunohistochemistry

Ovaries and imaginal discs were dissected in PBS, fixed for 20 min in 4% paraformaldehyde in PBS, washed for 30 min in PBS/0.1% Triton X-100 (PBST) and blocked for 15 min in 5% normal goat serum/PBST (PBST/NGS). Primary antibodies were diluted in PBST/NGS and samples were incubated overnight at 4°C.

Primary antibodies used were: mouse anti-Cut (1:100, DSHB), mouse anti-β-gal (Promega, 1:500), rabbit anti-expanded (1:200, a gift from A. Laughon, University of Wisconsin-Madison, USA), rabbit anti-PKCζ (C-20) (1:250, Santa Cruz), rat anti-Crumbs (1:300, a gift from U. Tepass, University of Toronto, Canada), mouse anti-Dlg (1:250, DSHB), rabbit anti-Kibra (1:200; Genevet et al., 2010), rabbit anti-Src pY418 (1:150, Life Tech), FITC-conjugated anti-GFP (1:400, Abcam), rabbit anti-βH-Spectrin (1:100, a gift from G. Thomas, Pennsylvania State University, USA), guinea pig anti-Merlin (1:100, a gift from R. Fehon, University of Chicago, IL, USA), mouse anti-eyes absent (1:250, DSHB) and mouse anti-Coracle (1:100).

Secondary antibodies (all from Molecular Probes or Invitrogen) were used at 1:500 for 2-4 h prior to multiple washes in PBST and staining with DAPI at 1 mg/ml for 10-30 min prior to mounting on slides in Vectashield (Vector labs).

### Image acquisition

Confocal images were taken with a Leica SP5 confocal microscope using 20× and 40× oil immersion objectives, and processed with Adobe Photoshop and Fiji. Either optical cross-sections through the middle of the different tissues or the top focal plane are shown in all figures. Pictures of the adult wings mounted in Hoyer's medium were taken on a Zeiss Axioplan 2 Imaging Microscope.

### Quantification of fluorescence intensity

For *dic* and *wts* clones, the ratio or nuclear to cytoplasmic Yki:GFP in a cell was calculated by drawing freehand ROIs in ImageJ in the respective compartments on cross-sections of confocal images. The ROI measurement tool was then used to calculate the mean grey values for each area and the ratio was calculated.

For measuring nuclear to cytoplasmic Yki:GFP in relation to cell shape changes, cross-section confocal images were taken and each cell was measured for height and width using ImageJ. Then for the same cell, the nuclear:cytoplasmic ratio of Yki:GFP (described above) was plotted against its cell height:width ratio. An exponential trend line was fitted using Excel.

The same analysis was performed for Kib:GFP with the exception that, instead of using the freehand ROI tool, the signal from the apical membrane was measured using the linear ROI measurement tool. A linear trend line was fitted using Excel.

## Supplementary Material

Supplementary information

Supplementary information
