# Supplementary material for: Mechanical strain regulates the Hippo pathway in Drosophila
Source: Development. 2018 Mar 1;145(5):dev159467. doi: 10.1242/dev.159467 (PMC5868995; doi:10.1242/dev.159467)
Supplement: Supplementary information [file develop-145-159467-s1.pdf]

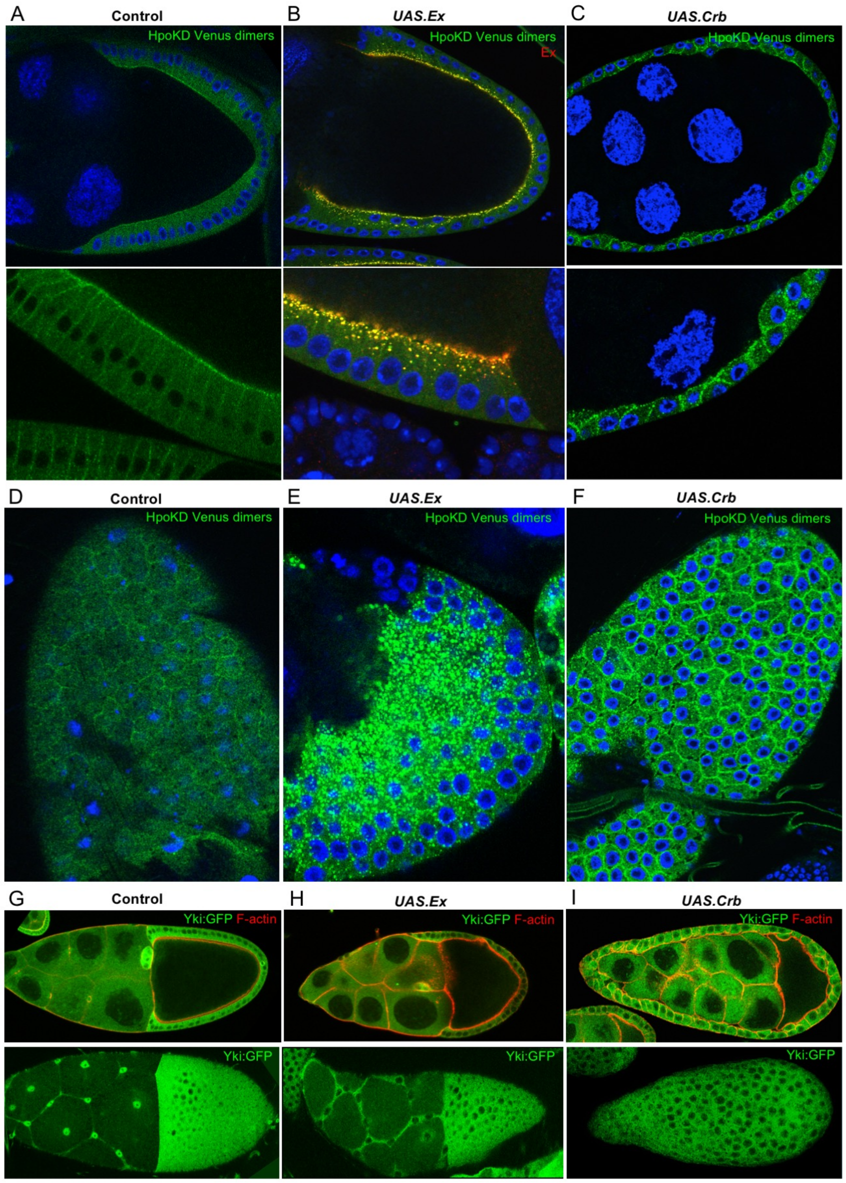


**Figure S1. Ectopic induction of Hippo dimerisation and signalling to Yki upon overexpression of Expanded or Crumbs.**

(A, D) Expression of split-Venus Hippo dimerisation sensor in the follicular epithelium reveals strong apical activation in columnar follicle cells.

(B, E) Overexpression of Expanded further induces Hippo-Venus dimerisation and induces internalisation into punctae.

(C, F) Overexpression of Crumbs further induces Hippo-Venus dimerisation and induces spreading of Hippo complexes around the plasma membrane due to an expansion of the apical domain.

(G) Yki:GFP in a control stage 10 egg chamber

(H) Yki:GFP in a stage 10 egg chamber expressing *UAS.Ex* under *TJ.Gal4.*

(I) Yki:GFP in a stage 10 egg chamber expressing *UAS.Crb* under *TJ.Gal4*.

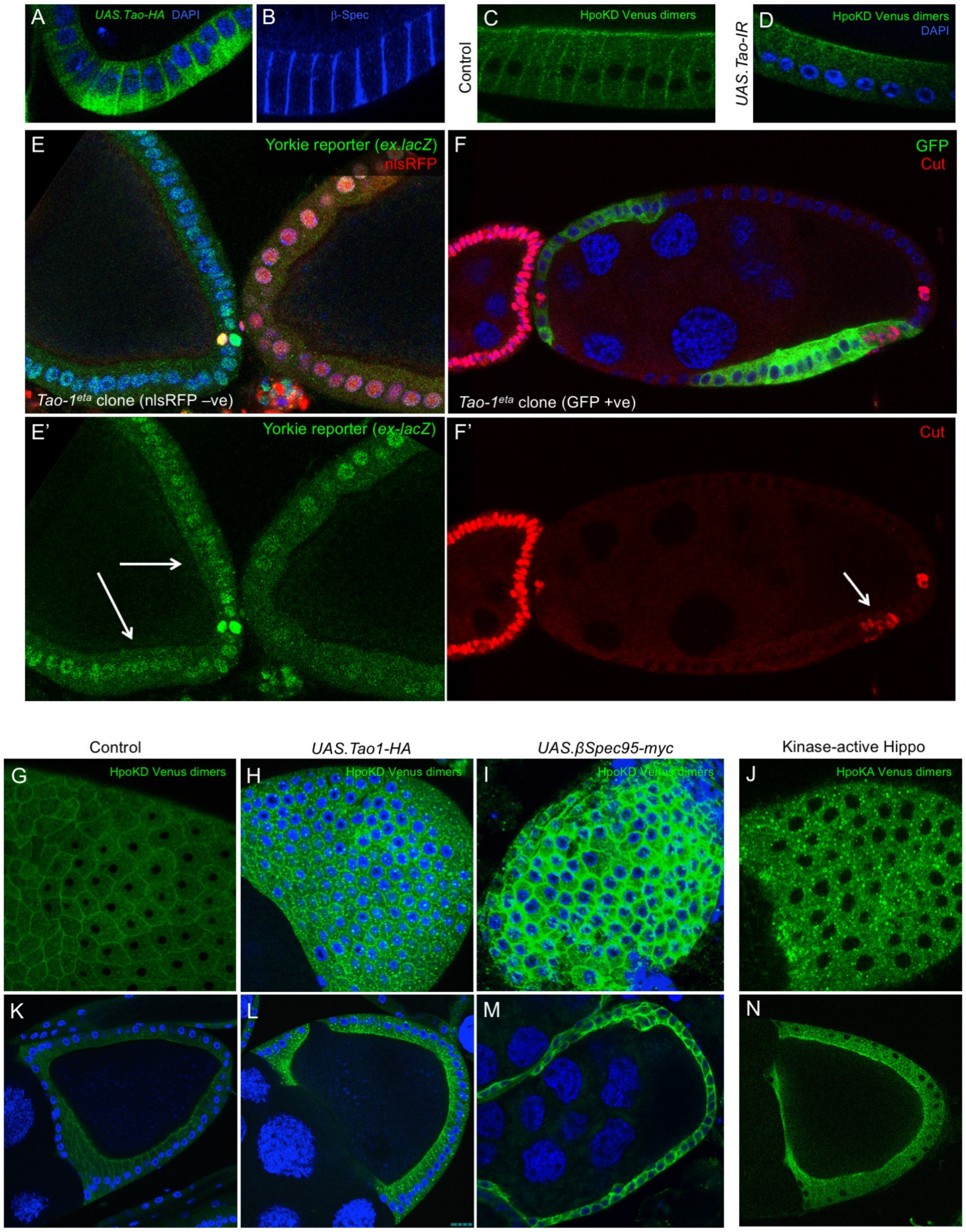


**Figure S2. Tao-1 localises laterally with beta-Spectrin to activate Hippo dimerisation and help repress Yorkie target genes in posterior follicle cells.**

(A,B) HA-tagged Tao-1 localises laterally with beta-Spectrin in columnar follicle cells.
(C,D) Silencing of Tao-1 by expression of *UAS.Tao1-RNAi* with the *GR1.Gal4* driver causes a reduction in lateral Hippo-Venus dimerisation signal, without affecting the apical signal.
(E) Mutation of Tao-1 in clones marked by the absence of nuclear RFP (red) lead to increased levels of *ex.lacZ* expression in columnar follicle cells.
(F) Mutation of Tao-1 in clones marked by the presence of cytoplasmic GFP (green) lead to increased levels of Cut expression in the posterior-most columnar follicle cells.
(G-N) Overexpression of Tao-1, beta-Spectrin, or kinase-active Hippo-Venus reporter further induces Hippo-Venus dimerisation at either the plasma membrane or intracellular punctae.

Development • Supplementary information

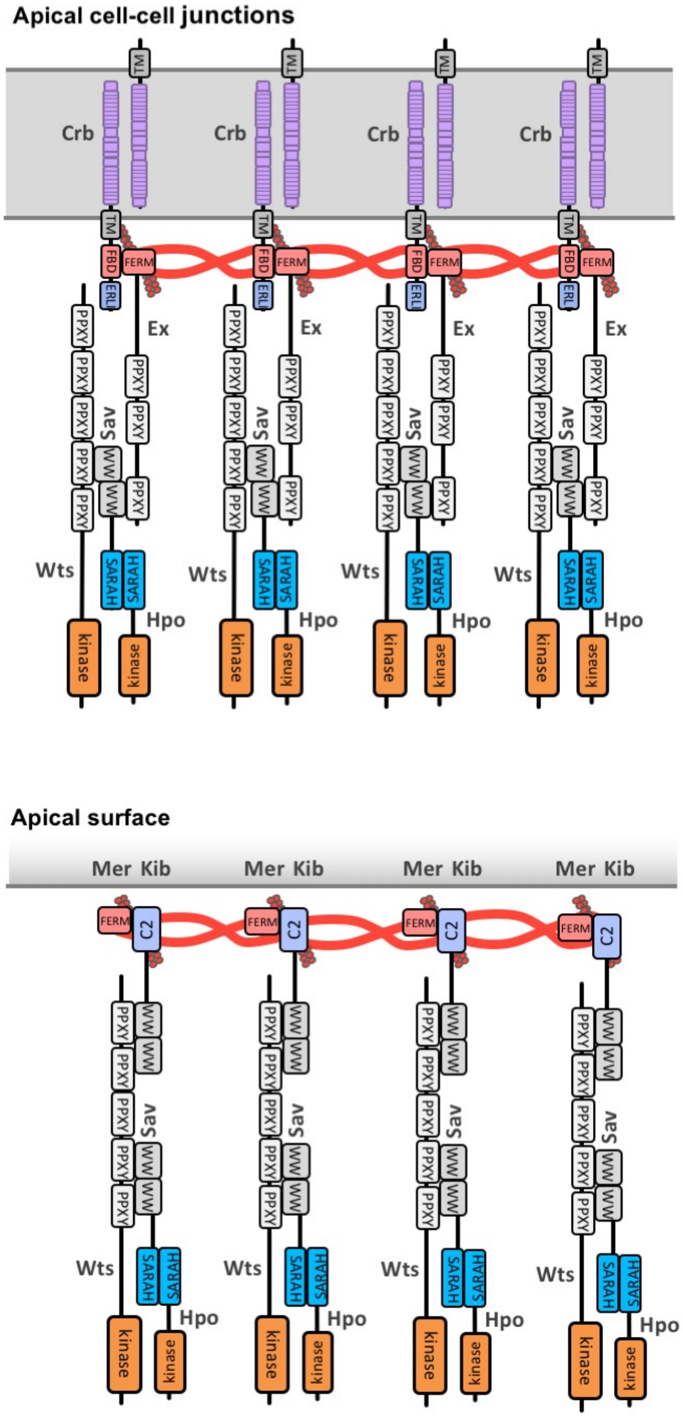


**Figure S3. Both apical-junctional Crb-Ex and apical-medial Mer-Kib complexes contribute to Hippo pathway regulation in follicle cells.**
Schematic model showing two pools of Hippo kinase regulation, one at the apical junction mediated by Crb and Ex and the other one at the apical-medial surface mediated by Mer and Kib.

Development • Supplementary information

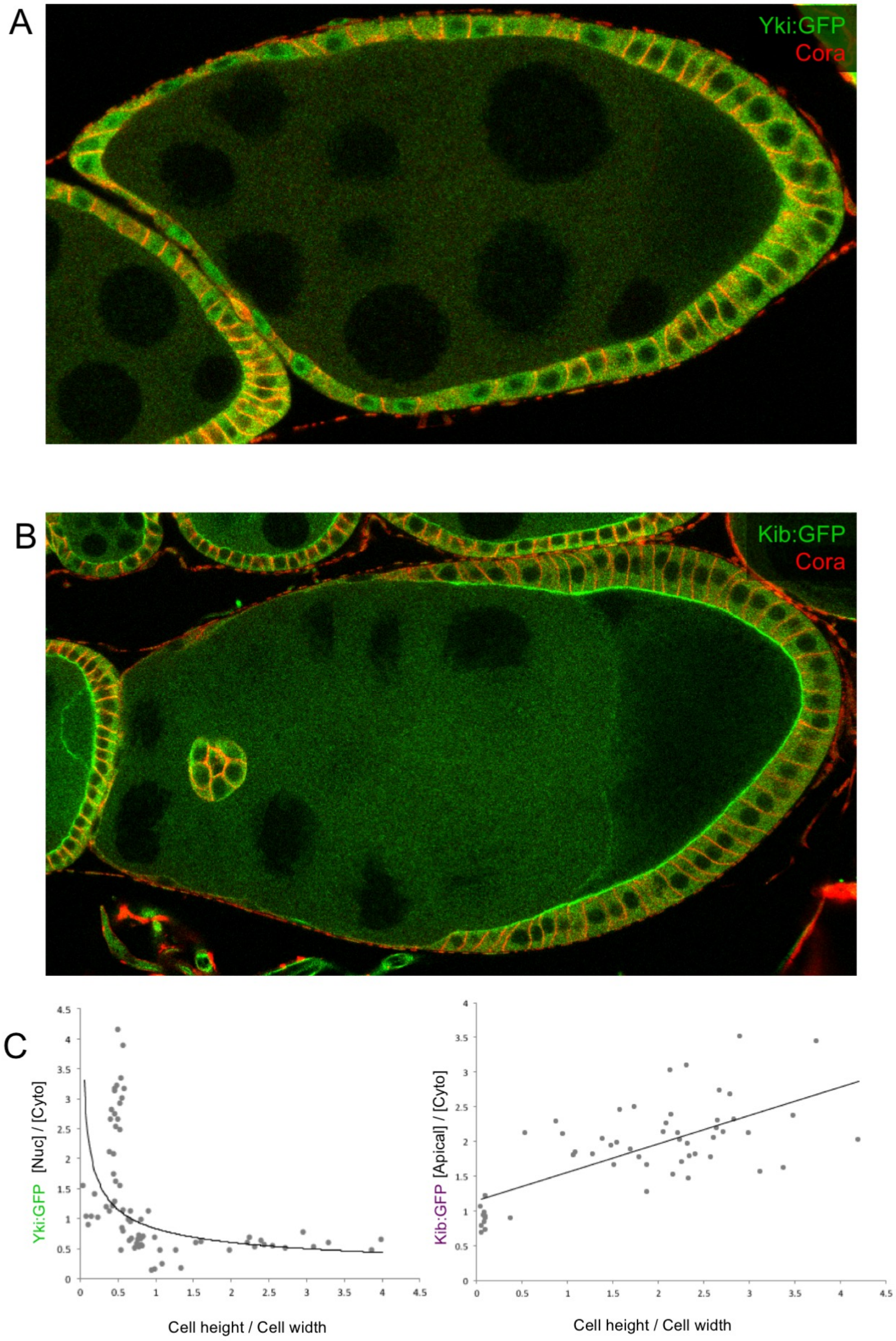


**Figure S4. Quantification reveals an exponential increase in Yki nuclear localisation upon linear dilution of the apical Hippo pathway component Kibra in stretch cells.**

(A) Stage 9 egg chamber expressing Yki:GFP. Coracle in red marks the lateral membrane. Dapi staining is shown in blue.

(B) Stage 9 egg chamber expressing Kib:GFP. Coracle in red marks the lateral membrane. Dapi staining is shown in blue.

(C) Quantification of nuclear:cytoplasmic ratio of Yki:GFP (n=72) and apical:cytoplasmic ratio of Kib:GFP (n=55) plotted against cell height relative to cell width.

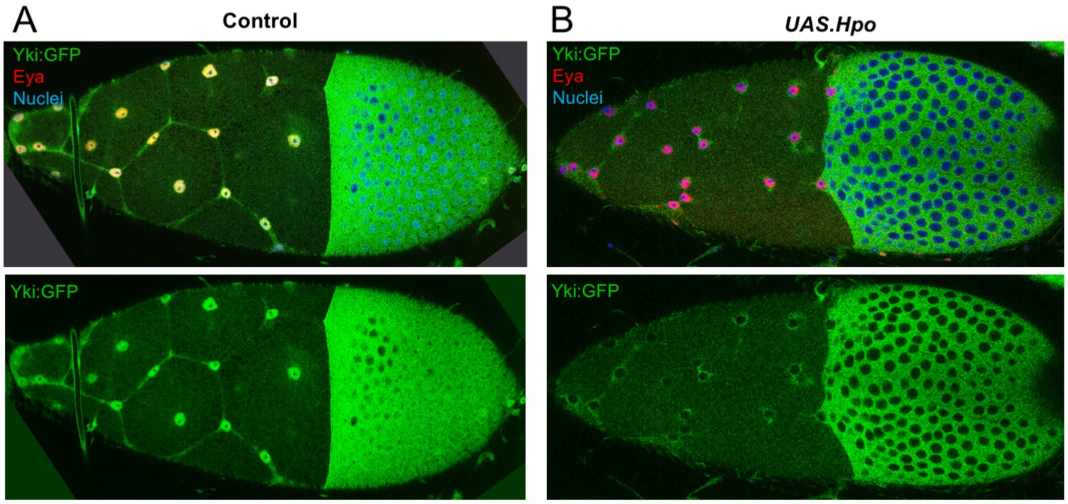


**Figure S5. Hippo activation and loss of nuclear Yki does not affect stretch cell fate specification, as measured by expression of the Eyes-absent (Eya) transcription factor.**
(A) Control stage 10 egg chamber expressing Yki:GFP (green) and stained for Eya (red) and Dapi (blue).
(B) TJ.Gal4 expressing *UAS.Hpo* results in loss of nuclear Yki:GFP (green) but does not affect Eya (red).

Development • Supplementary information

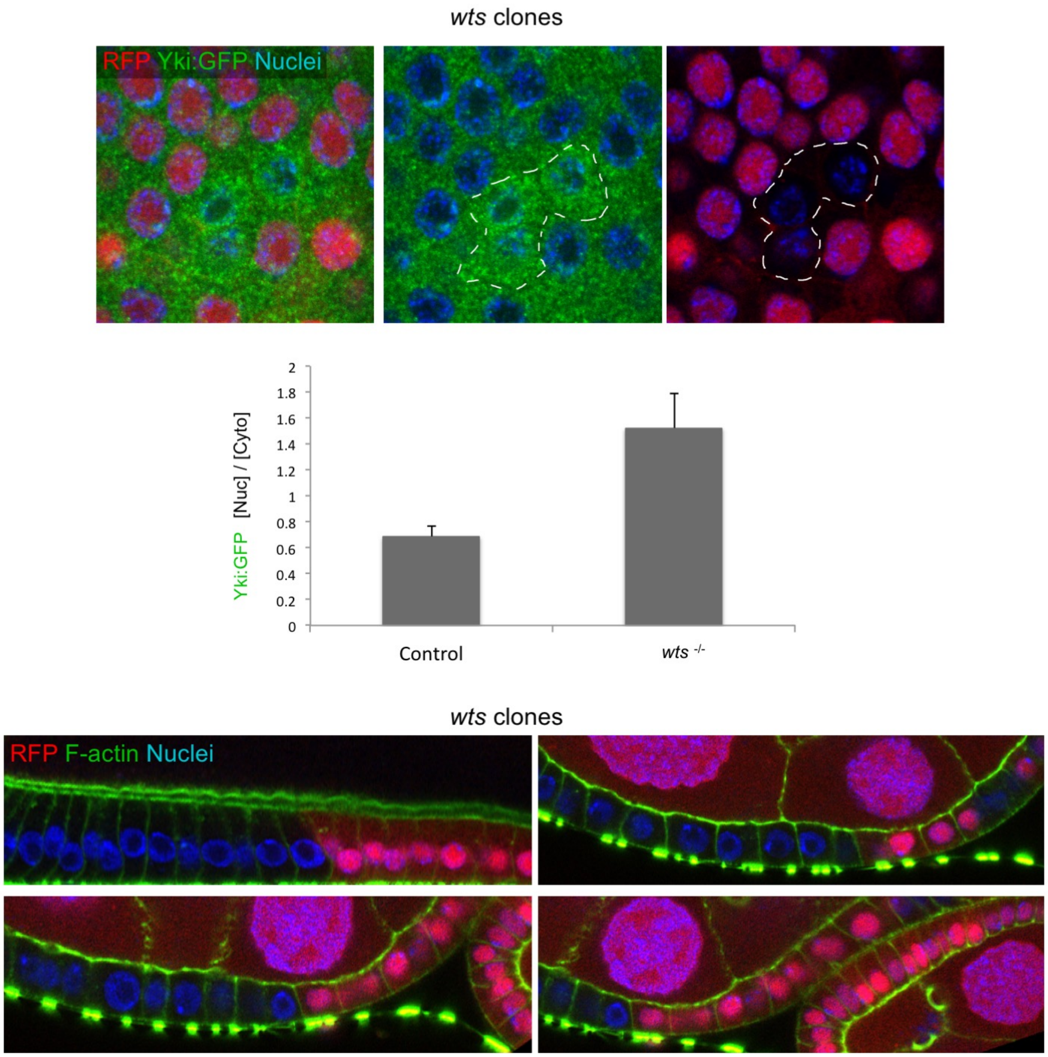


**Figure S6: *wts* clones induce nuclear Yki:GFP but do not affect positioning of the nucleus.** Quantification of nuclear:cytoplasmic ratio of Yki:GFP in *wts* clones (top; RFP negative cells, n=6). Average and standard deviation (error bars) are represented. Cross sections through different stage egg chambers containing *wts* clones showing that nuclear positioning (DAPI, blue) does not change (bottom).

Development • Supplementary information

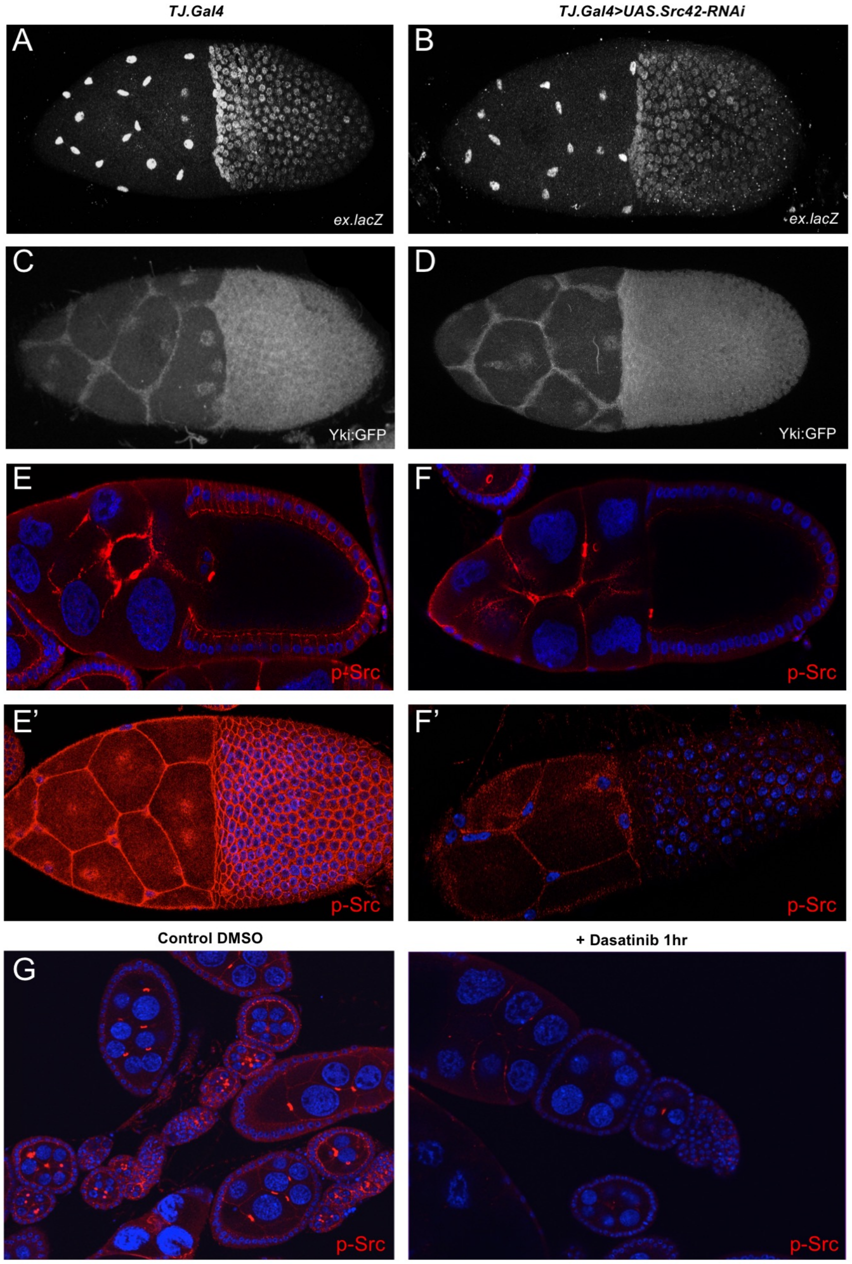


**Figure S7. Inhibition of Src with RNAi or Dasatanib treatment reduces p-Src levels but does not affect Yki:GFP localisation or ex.lacZ expression.**

(A) Control stage 10 *Ex.LacZ* egg chamber

(B) Silencing of Src42a by *UAS.RNAi* hairpin expression with TJ.Gal4 driver does not reduce expression of ex.lacZ in stretch cells.

(C) Control stage 10 Yki:GFP egg chamber

(D) Silencing of Src42a by *UAS.RNAi* hairpin expression with TJ.Gal4 driver does not reduce nuclear localisation Yki:GFP in stretch cells.

(E,F) Cross-section and apical views of phospho-Src staining shows the reduction in active Src levels upon *Src42a-RNAi*.

(G) Dasatinib is an effective inhibitor of Src activity as measured by autophosphorylation.

Development • Supplementary information

Dapi staining is shown in blue.

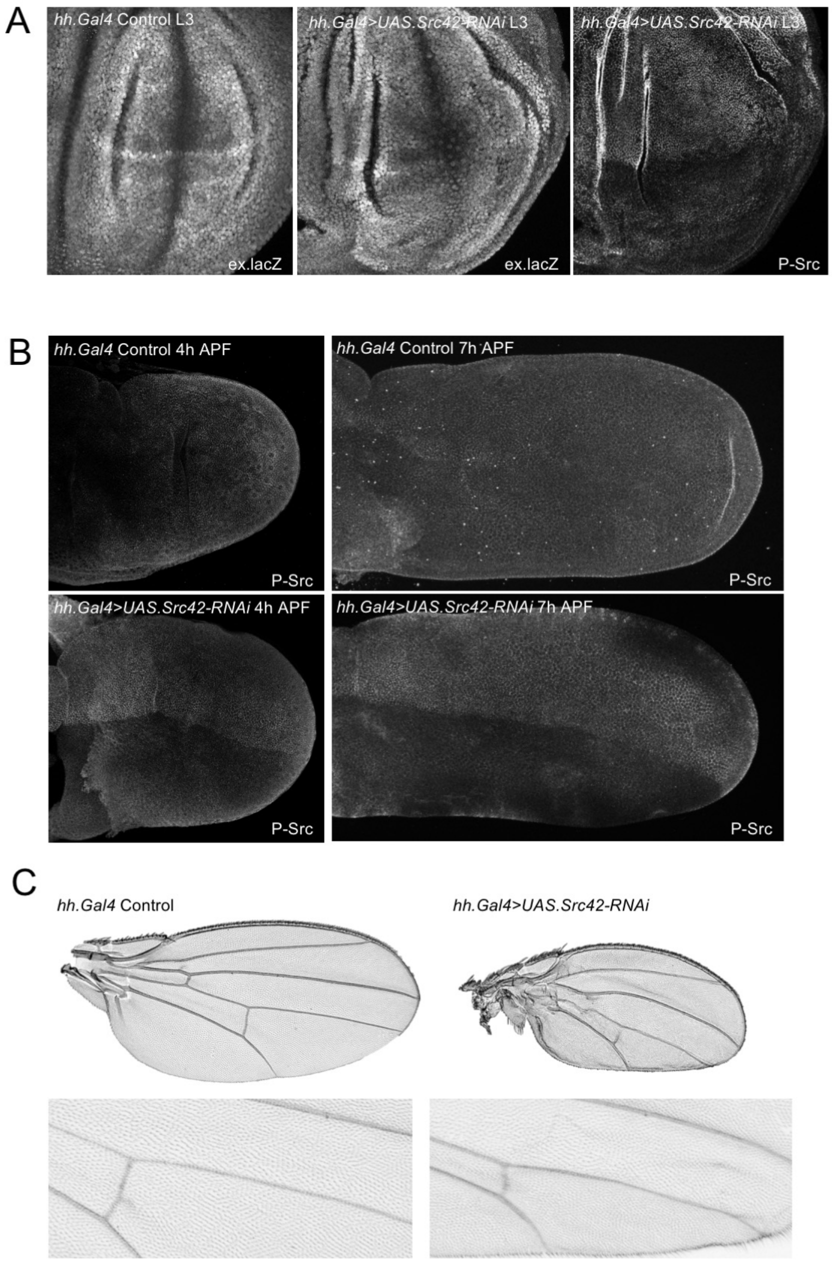


**Figure S8. Src42 RNAi does not affect Yki target gene expression but does reduce cell size in the wing, indicating a Hippo pathway independent function.**
(A) Silencing of Src42 by *UAS.RNAi* hairpin expression with *Hedgehog-Gal4* (*hh.Gal4*) driver does not reduce expression of ex.lacZ in the wing imaginal disc.
(B,C) Silencing of Src42 by *UAS.RNAi* hairpin expression with *hh.Gal4* driver reduces posterior compartment size and cell size (revealed by increased hair density) adult wings.

Development • Supplementary information

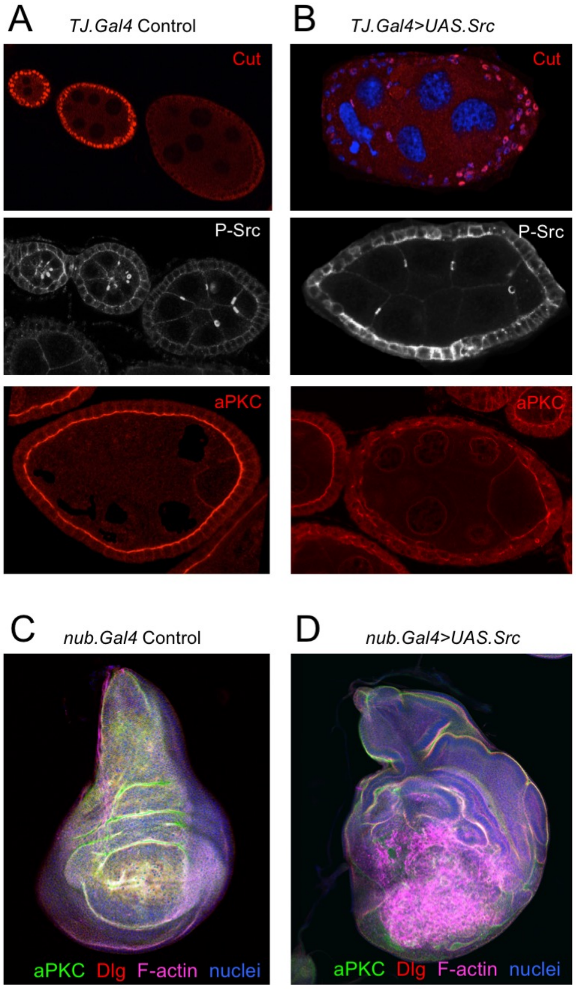


**Figure S9. Overexpression of Src indirectly affects Yki target gene expression via alterations in epithelial cell polarity.**
(A,B) Overexpression of *UAS.Src* with the *TJ.Gal4* driver induces expression of the Yki target gene Cut, but also disrupts the apical domain of follicle cells at stage 8 of oogenesis.
(C,D) Overexpression of *UAS.Src* with the nubbin.Gal4 (nub.Gal4) driver disrupts epithelial cell polarity in the wing imaginal disc, suggesting an indirect effect of Src overexpression on Yki.

Development • Supplementary information

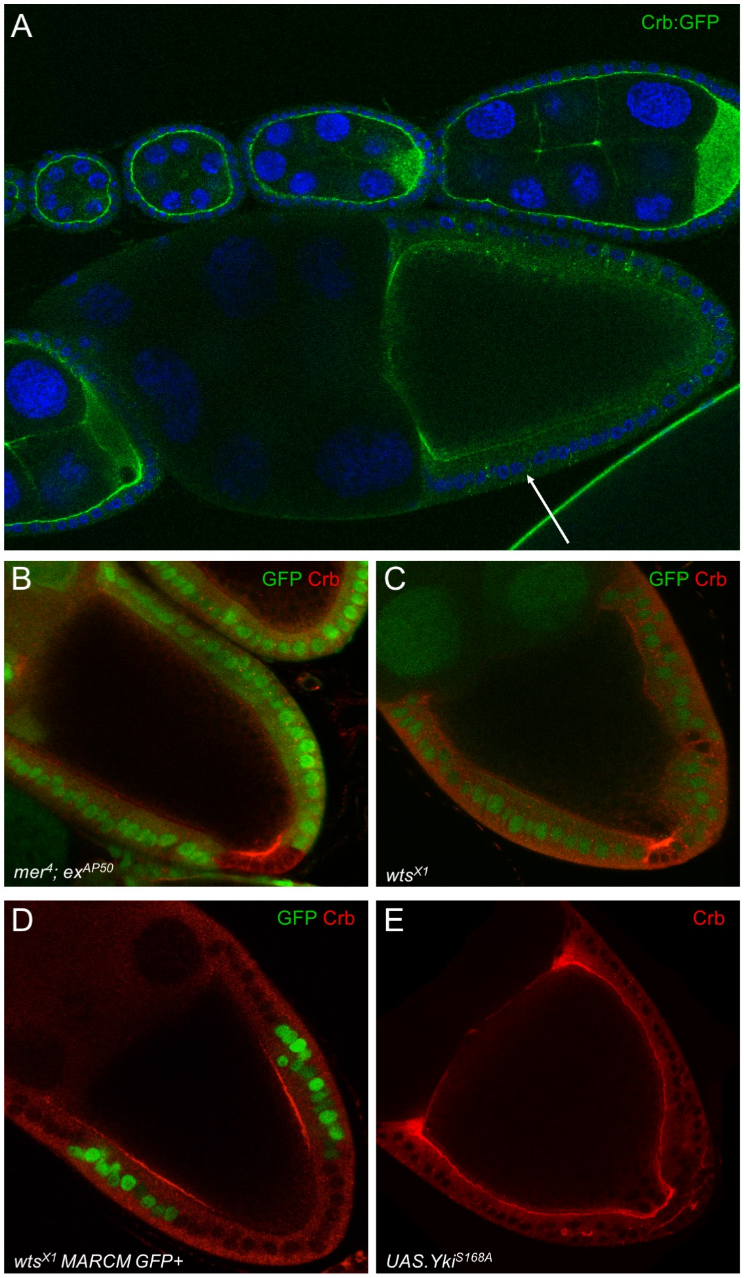


**Figure S10. Activation of Hippo signalling in columnar cells at stage 10 of oogenesis downregulates Crb in a negative feedback loop.**

(A) Crb:GFP is strongly expressed at the apical membrane of follicle cells prior to stage 10 after which it becomes clearly down-regulated.

(B) *mer, ex* double clones (GFP negative) have increased levels of Crb at the apical membrane.

(C) *wts* clones (GFP negative) have increased levels of Crb at the apical membrane.

(D) *wts* clones (GFP positive) have increased levels of Crb at the apical membrane.

(E) *TJ.Gal4, UAS-Yki*$^{S168A}$ expressing stage 10 egg chamber in which Crb remains at the apical membrane in all follicle cells.

Development • Supplementary information

## Table S1. *Drosophila* genotypes

Fig S1A, D: *TJ.Gal4, UAS.Hippo*$^{Kinase\text{-}dead}$*VenusC; UAS.Hippo*$^{Kinase\text{-}dead}$*VenusN/+*
Fig S1B, E: *TJ.Gal4, UAS.Hippo*$^{Kinase\text{-}dead}$*VenusC; UAS.Hippo*$^{Kinase\text{-}dead}$*VenusN/UAS-Ex; +*
Fig S1C, F: *TJ.Gal4, UAS.Hippo*$^{Kinase\text{-}dead}$*VenusC; UAS.Hippo*$^{Kinase\text{-}dead}$*VenusN/UAS-Crb; +*
Fig S1G: *GR1.Gal4, Yki:GFP/+*
Fig S1H: *GR1.Gal4, Yki:GFP/UAS.Ex*
Fig S1I: *GR1.Gal4, Yki:GFP/UAS.Crb*

Fig S2A: *TJ.Gal4/UAS.Tao-HA* (Gomez et al., 2012)
Fig S2B: *TJ.Gal4/UAS.Tao-HA*
Fig S2C: *TJ.Gal4, UAS.Hippo*$^{Kinase\text{-}dead}$*VenusC; UAS.Hippo*$^{Kinase\text{-}dead}$*VenusN/+*
Fig S2D: *TJ.Gal4, UAS.Hippo*$^{Kinase\text{-}dead}$*VenusC; UAS.Hippo*$^{Kinase\text{-}dead}$*VenusN/+; UAS.TaoIR (VDRC KK107645)*
Fig S2E: *hs.flp, frt19a ubiRFPnls/Tao*$^{eta}$*19a; ex*$^{lacz}$*/+*
Fig S2F: *hs.flp, frt19a ubiRFPnls/Tao*$^{eta}$*19a*
Fig S2G: *TJ.Gal4, UAS.Hippo*$^{Kinase\text{-}dead}$*VenusC; UAS.Hippo*$^{Kinase\text{-}dead}$*VenusN/+*
Fig S2H: *TJ.Gal4, UAS.Hippo*$^{Kinase\text{-}dead}$*VenusC; UAS.Hippo*$^{Kinase\text{-}dead}$*VenusN/UAS.Tao-HA; +*
Fig S2I: *TJ.Gal4, UAS.Hippo*$^{Kinase\text{-}dead}$*VenusC; UAS.Hippo*$^{Kinase\text{-}dead}$*VenusN/UAS.βSpec95-myc* (Mazock et al., 2010)
Fig S2J: *TJ.Gal4, UAS.Hippo*$^{Kinase\text{-}active}$*VenusC; UAS.Hippo*$^{Kinase\text{-}active}$*VenusN/+*
Fig S2K: *TJ.Gal4, UAS.Hippo*$^{Kinase\text{-}dead}$*VenusC; UAS.Hippo*$^{Kinase\text{-}dead}$*VenusN/+*
Fig S2L: *TJ.Gal4, UAS.Hippo*$^{Kinase\text{-}dead}$*VenusC; UAS.Hippo*$^{Kinase\text{-}dead}$*VenusN/UAS.Tao-HA; +*
Fig S2M: *TJ.Gal4, UAS.Hippo*$^{Kinase\text{-}dead}$*VenusC; UAS.Hippo*$^{Kinase\text{-}dead}$*VenusN/UAS.βSpec95-myc (Mazock et al., 2010)*
Fig S2N: *TJ.Gal4, UAS.Hippo*$^{Kinase\text{-}active}$*VenusC; UAS.Hippo*$^{Kinase\text{-}active}$*VenusN/+*

Fig S4A: *Yki:GFP*
Fig S4B: *Kib:GFP*

Fig S5A: *TJ.Gal4, Yki:GFP /+*
Fig S5B: *TJ.Gal4, Yki:GFP /UAS-Hippo*

Fig S6: *hsflp/+; Yki:GFP / Yki:GFP; wtsXI 82B/82B GFP*

Fig S7A: *ex-LacZ, TJ.Gal4/+*
Fig S7B: *ex-LacZ TJ.Gal4/+ UAS-Src42a.IR (VDRC 26019)/+*
Fig S7C: *Yki:GFP , TJ.Gal4/+*
Fig S7D: *Yki:GFP, TJ.Gal4/+; UAS-Src42a.IR (VDRC 26019)/+*
Fig S7E, E': *TJ.Gal4*
Fig S7F, F': *TJ.Gal4/+ UAS-Src42a.IR (VDRC 26019)/+*
Fig S7G: *W*$^{iso}$

Fig S8A: *ex-LacZ; Hh.G4/+* and *ex-LacZ; Hh.G4/UAS-Src42a.IR*
Fig S8B: *Hh.G4/+* and *Hh.G4/UAS-Src42a.IR*
Fig S8C: *Hh.G4/+* and *Hh.G4/UAS-Src42a.IR*

Fig S9A: *TJ.Gal4/+*
Fig S9B: *TJ.Gal4/UAS.Src64b*
Fig S9C: *Nub.G4/+*
Fig S9D: *Nub.G4/UAS.Src64b*

Fig S10A: *Crb:GFP*
Fig S10B: *hsflp 19a GFP/19a* $mer^{4}$, $ex^{AP50}$
Fig S10C: *hsflp;; FRT82B* $wts^{XI}$*/FRT82B GFP*
Fig S10D: *yw TubGAL4 hsFLP 122 UAS-nucGFPmyc;; FRT82B CD21 y+ TubG80.LL3/FRT82B* $wts^{XI}$
Fig S10E: *TJ.Gal4/UAS-*$Yki^{S168A}$*; Crb:GFP/+*

## References

**Gomez, J. M., Wang, Y. and Riechmann, V.** (2012). Tao controls epithelial morphogenesis by promoting Fasciclin 2 endocytosis. *J Cell Biol* **199**, 1131-1143.

**Mazock, G. H., Das, A., Base, C. and Dubreuil, R. R.** (2010). Transgene rescue identifies an essential function for Drosophila beta spectrin in the nervous system and a selective requirement for ankyrin-2-binding activity. *Mol Biol Cell* **21**, 2860-2868.
